# Hip Fracture in the Elderly: A Re-Analysis of the EPIDOS Study with Causal Bayesian Networks

**DOI:** 10.1371/journal.pone.0120125

**Published:** 2015-03-30

**Authors:** Pascal Caillet, Sarah Klemm, Michel Ducher, Alexandre Aussem, Anne-Marie Schott

**Affiliations:** 1 Hospices Civils de Lyon, Pôle Information Médicale Evaluation Recherche, Lyon, France; 2 LIRIS UMR 5205 CNRS, Data Mining & Machine Learning (DM2L) Team, Université Claude Bernard Lyon 1, Bâtiment Nautibus, Villeurbanne, France; 3 Hospices Civils de Lyon, Groupement Hospitalier de Gériatrie, Francheville, France; 4 Université de Lyon, Université Lyon 1, Lyon, France; 5 INSERM U1033, Lyon, France; Baylor College of Medicine, UNITED STATES

## Abstract

**Objectives:**

Hip fractures commonly result in permanent disability, institutionalization or death in elderly. Existing hip-fracture predicting tools are underused in clinical practice, partly due to their lack of intuitive interpretation. By use of a graphical layer, Bayesian network models could increase the attractiveness of fracture prediction tools. Our aim was to study the potential contribution of a causal Bayesian network in this clinical setting. A logistic regression was performed as a standard control approach to check the robustness of the causal Bayesian network approach.

**Setting:**

EPIDOS is a multicenter study, conducted in an ambulatory care setting in five French cities between 1992 and 1996 and updated in 2010. The study included 7598 women aged 75 years or older, in which fractures were assessed quarterly during 4 years. A causal Bayesian network and a logistic regression were performed on EPIDOS data to describe major variables involved in hip fractures occurrences.

**Results:**

Both models had similar association estimations and predictive performances. They detected gait speed and mineral bone density as variables the most involved in the fracture process. The causal Bayesian network showed that gait speed and bone mineral density were directly connected to fracture and seem to mediate the influence of all the other variables included in our model. The logistic regression approach detected multiple interactions involving psychotropic drug use, age and bone mineral density.

**Conclusion:**

Both approaches retrieved similar variables as predictors of hip fractures. However, Bayesian network highlighted the whole web of relation between the variables involved in the analysis, suggesting a possible mechanism leading to hip fracture. According to the latter results, intervention focusing concomitantly on gait speed and bone mineral density may be necessary for an optimal prevention of hip fracture occurrence in elderly people.

## Introduction

Hip fractures commonly result in permanent disability, institutionalization or death, and are one of the most damaging fractures among elderly people [1]. As the cost of fracture regarding medical expenditures and quality of life lost can be substantial, it is essential to identify a complete profile of fracture risk for the development of timely interventions such as pharmacotherapy to limit bone structure degradation and then prevent its clinical translation into hip fracture [2]. This degradation remains often definitive, i.e. it can be stopped but cannot be healed in most of the case [3]. Several tools exist to help clinician in the prediction and prevention of hip fractures [4]. However, their utility in clinical practice is debated and some studies showed that they are underused by practitioners [5]. One of the barriers for their use is their generally poor graphic presentation, which does not permit either to understand the underlying mechanisms or use the tool as an aid in explaining them to the patients [6]. Graphical models like Bayesian networks show an increasing popularity in the biomedical domain [7–12]. The graphical part of this type of model is very expressive for a modelling non-specialist and their implementation in existing scores could potentially contribute to their use in clinical practice. However, their potential contribution to the regression modelling approach needs to be studied and assessed before proceeding to such implementation. Our objective was to use a causal Bayesian network framework for studying mechanisms leading to hip fracture and our secondary objective was to confirm our results by performing a logistic regression.

## Methods

### Study population

For the purpose of this study, we used the EPIDOS cohort lastly updated in 2010 and already described elsewhere [4]. Briefly, 7598 women aged 75 years or older were recruited in five French cities (Amiens, Lyon, Montpellier, Paris, and Toulouse) and followed up by mailed questionnaires every 4 months during 4 years. Women who were not able to walk independently and those who had a bilateral hip replacement were excluded. Femoral-neck BMD by dual-photon X-ray absorptiometry, potential risk factors for osteoporosis and potential fall-related risk factors were assessed, which included self-reported physical capacity, neuromuscular function, mobility, visual function, history of previous falls and use of medication. During an average of 3.8 years of follow-up, 293 women suffered a hip fracture. After this 4 year period, only the vital status was regularly assessed until 2010 by checking the French national registry of death (INSEE). This study has been specifically approved by the French ethic committee 'Comité consultatif de protection des personnes dans la recherche biomédicale de Lyon B' in January 1992.

Based on literature, especially on the FRAX tool regarding risk factors for osteoporosis [13] and on expert knowledge, we used a set of 15 variables to describe the study population: age, body mass index at inclusion, current or past use of corticoids during 3 months or more, t-score at femoral neck, number of falls during the 6 months before inclusion, weekly intake of alcohol, tobacco smoking status, history of hip fracture since 55 of age, parental history of hip fracture, gait speed, Five Times Sit To Stand test results (5TSTS) which is a proxy of the motor performances of the patients [14], number of recorded chronic diseases (diabetes, depression, glaucoma, cataract, angina pectoris, Parkinson disease and hypertension), current or past use of vitamin D in the past year, current psychotropic drug use and hip fracture. Hip fractures were ascertained by X-rays radiography and analyzed by an expert rheumatologist. Data were discretized when needed according to expert knowledge and another analysis using EPIDOS data [15]. For the comparison purpose of the study, we used the same dataset for each modeling approach.

### Statistics

**Fig 1:**
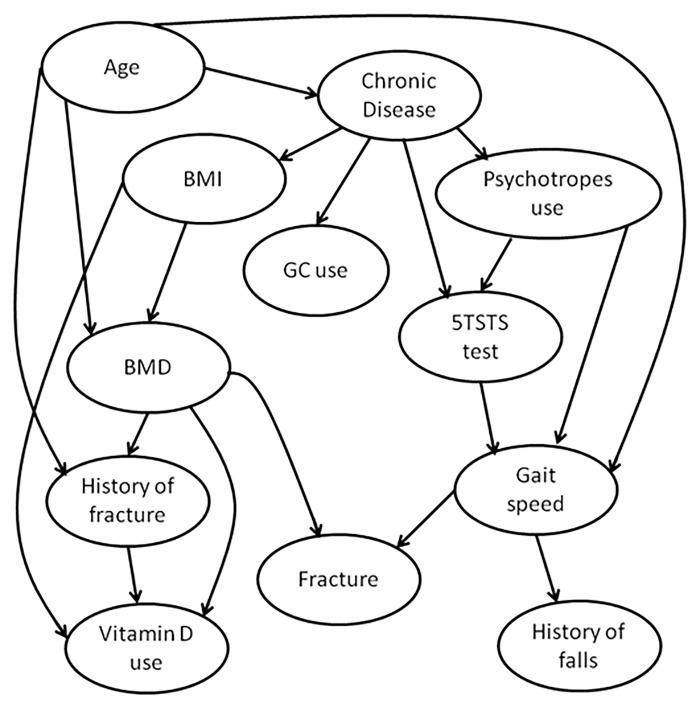
Causal Bayesian network structure.

In this study, we used a hybrid algorithm, called H2PC, to learn the Bayesian network (BN) structure among the 15 candidate variables discussed above. The source code of H2PC and the Bnlearn package in R [16] are publicly available. The set of causal assumptions used in this study is described in Table 1. The resulting DAG is interpreted as causal BN. The bootstrapping process has been repeated 200 times and an averaged DAG representing the final Bayesian network was drawn, containing only arcs appearing in at least 25% of the DAGs constructed earlier from the bootstrapped datasets (Fig 1). Analysis was performed using R software (v.2.13.0) and Netica Software (v.4.16, Norsys Software Corp, Vancouver, Canada). A more comprehensive description of the Bayesian network modelling approach can be found in many published work [17,18].

**Table 1:** Logical constraints applied on the structural learning stage.

	Age	BMI	BMD	Gait speed	5STST	History of fracture	Parental history of fracture	Chronic diseases	Vitamin D use	GC use	Psychotropes use	Alcohol	Tobacco smoking	History of fall	Hip fracture
Age		■	■	■	■	■	■	■	■	■	■	■	■	■	■
BMI			■	■										■	■
BMD						■								■	■
Gait speed			■											■	■
5STST			■	■											■
History of fracture															■
Parental history of fracture	■	■	■	■	■	■		■	■	■	■	■	■	■	■
Chronic diseases			■	■	■						■				■
Vitamin D use															■
GC use									■						■
Psychotropes use				■											■
Alcohol			■	■	■		■			■				■	■
Tobacco smoking			■	■	■		■			■				■	■
History of fall			■										■		■
Hip fracture															

A black square means “cannot be directed torwards”. For example, in the “Age” column, presence of a square in the “Parental history of fracture” line encodes the assumption that parental history of fracture cannot be directed torwards the age of the patient.

We used a logistic regression model with a stepwise selection approach (retained threshold of p<0.20 for prior inclusion, and p>0.05 for exclusion of variables in the model, which are common thresholds in epidemiology). Continuous variables were discretized prior to inclusion in the model according to the expert. Patients presenting missing values (2% of the initial sample) were excluded from the analysis (complete case approach). All the covariates had a number of events greater than 10 and the outcome variable counted more than 200 events. Interactions were tested with a threshold of p<0.05, in case of interaction a stratified analysis was conducted. Collinearity between variables was checked with the approach of Belsley, Kuh, and Welsch [19] (a threshold of 30 was chosen for collinearity detection). A test of Hosmer and Lemeshow goodness-of-fit was performed [20], with a retained threshold for statistical significancy retained at p = 0.05. Analysis was performed using SAS software (v.9.3, SAS Institute Inc., CARY, NC, USA).

A receiver operating characteristic (ROC) curve was plotted to evaluate each model predictive performances [21]. The area under the ROC curve (AUROC) was then calculated for both logistic regression and BN to assess their overall performances regarding fracture prediction. Each ROC curve was compared using a contrast test [22]. Sensibility, specificity, positive and negative likelihood ratios and positive and negative predicted value were computed, considering the respective Youden index [23] for each model.

## Results

7547 women were included in this analysis (51 patients were excluded due to missing values). Characteristics of the population are shown in Table 2. The mean age was 80.5 years (SD = 3.8). A total of 289 patients sustained a fracture during the follow-up. Relationships between potential risk factors and fracture were assessed independently by the two approaches (i.e logistic regression and causal Bayesian network) and results are displayed in Table 3.

**Table 2:** Characteristics of the study patients at inclusion (n = 7547).

Variable (name in the graph)	N (%)
**Age**	
<80 y	3960 (52.5)
80-<85 y	2623 (34.8)
85-<90 y	832 (11.0)
> = 90 y	132 (1.70)
**BMI**	
<18.5	220(2.90)
18.5–30	6361 (84.3)
>30	966 (12.8)
**Gait speed**	
<0.6 m.s^-1^	979 (13.0)
0.6-<0.85 m.s^-1^	2668 (35.3)
0.85-<1 m.s^-1^	1968 (26.1)
> = 1 m.s^-1^	1932 (25.6)
**Alcool consumption**	
0–20g/week	6335 (83.9)
>20g/week	1212 (16.1)
**Tobacco smoking**	
Never	6493 (86.0)
Former	801 (10.6)
Actual	253 (3.40)
**History of fracture since 55 of age (history of fracture)**	
No	5534 (73.3)
Yes	2013 (26.7)
**History of parental fracture**	
No	6872 (91.1)
Yes	675 (8.90)
**More than 2 fall in the previous 6 months before inclusion (history of falls)**	
No	7316 (96.9)
Yes	231 (3.10)
**Number of current chronic diseases (Chronic disease)**	
0–1	2182 (28.9)
> = 2	5365 (71.1)
**Five Sit To Stand Test results (5STST)**	
1-<16s	4590 (60.8)
16s-<23s	1901 (25.2)
>23s	916 (12.1)
Incapacity	140 (1.80)
**Use of corticotherapy or history of corticotherapy> 3 months before inclusion (GC use)**	
No	7303 (96.7)
Yes	251 (3.30)
**Current use of vitamin D or history of use of vitamin D in the past year before inclusion (vitamin D use)**	
No	6484 (85.9)
Yes	1063 (14.1)
**Current use of sedative or anxiolytics (Psychotropes use)**	
No	3516 (46.6)
Yes	4031 (53.4)
**Bone Mineral Density (BMD)**	
T-score >-2.5 SD	1955 (25.9)
T-score <-2.5 SD	5592 (74.1)
**Presence of hip fracture during the 4 year follow-up (Fracture)**	
No	7258 (96.2)
Yes	289 (3.80)

**Table 3:** Results of logistic regression and causal Bayesian network modeling (n = 7547).

	Final Multivariate Logistic regression		Bayesian Network	
Variable	ORa (95% CI)	p*	OR	Predicted probability of fracture^&^
**Current use of sedative or anxiolytics**				
No	Reference		Reference	0.0344
Yes	1.32 (1.02–1.69)	0.032	1.22	0.0418
**Use of corticotherapy or history of corticotherapy> 3 months**				
No	/	/	Reference	0.0384
Yes	/	/	1.01	0.0388
**Current use of vitamin D or history of use of vitamin D in the past year**				
No	/	/	Reference	0.0380
Yes	/	/	1.07	0.0408
**Number of current chronic diseases**				
0–1	/		Reference	0.0355
> = 2	/	/	1.11	0.0395
**History of parental fracture**				
No	/	/	/	/
Yes	/	/	/	/
**Five Sit To Stand Test (5STST)**				
1-<16s	/	/	Reference	0.0312
16s-<23s	/	/	1.42	0.0439
>23s	/	/	1.94	0.0588
Incapacity	/	/	2.11	0.0637
**Age (years)**				
<80	Reference		Reference	0.0347
80-<85	1.42 (1.07–1.88)	0.016	1.15	0.0397
85-<90	2.39 (1.71–3.34)	<0.0001	1.41	0.0482
> = 90	3.45 (1.95–6.12)	<0.0001	1.79	0.0604
Bone Mineral Density**				
T-score >-2.5 SD	Reference		Reference	0.0122
T-score <-2.5 SD	3.54 (2.29–5.46)	<0.0001	4.03	0.0475
**BMI**				
<18.5	1.67 (0.98–2.87)	0.06	1.13	0.0441
18.5–30	Reference		Reference	0.0390
>30	0.64 (0.42–0.98)	0.04	0.84	0.0332
Gait speed**				
> = 1 m.s^-1^	Reference		Reference	0.0146
0.85-<1 m.s^-1^	1.69 (1.06–2.71)	0.004	1.76	0.0254
0.6-<0.85 m.s^-1^	3.04 (1.99–4.64)	<0.0001	3.42	0.0483
<0.6 m.s^-1^	4.71 (2.99–7.43)	<0.0001	6.20	0.0841
**Alcool consumption**				
0–20g/week	/	/	/	/
>20g/week	/	/	/	/
**Tobacco smoking**				
Never	/	/	/	/
Former	/	/	/	/
Actual	/	/	/	/
**History of fracture since 55 of age**				
No	Reference		Reference	0.0381
Yes	1.39 (1.06–1.80)	0.014	1.03	0.0391
**More than 2 fall in the previous 6 months**				
No	Reference		Reference	0.0381
Yes	1.86 (1.10–3.14)	0.020	1.21	0.0459

^/^ Variable not included in the final model.

* P<0.05 = statistically significant.

** Variable directly linked to hip-fracture given the graph.

^&^ Predicted probability of fracture according to the causal Bayesian network model.

The most probable Bayesian network structure given our data was computed according to the observations and some *a priori* causal assumptions. These assumptions were encoded in a constraint matrix used in the learning process by the algorithmic approach and are shown in Table 1. In the causal pathways proposed by the final model (Fig 1), Age and BMI were found to influence directly the BMD and BMD was found to influence directly the probability of hip fracture occurrence. Psychotropic drug use was found to influence directly the gait speed and the 5 Times Sit To Stand (5TSTS) test results, which in turn influenced falls and fractures (Fig 1). Previous falls were not found to be directly associated with hip fracture in this model, despite the fact that the hypothesis of fall being a cause of fracture but not the opposite was allowed in the constraints used in the structure learning phase. The only two variables that were directly linked to fracture were gait speed and bone mineral density. The AUROC of our Bayesian network was 0.71, 95% Confidence Interval (95% CI) = (0.68–0.73). Both models gave similar prediction regarding hip fractures occurrences (Table 4), and had positive likelihood ratio about 2 and a negative likelihood ratio of 0.5 [24].

**Table 4:** Comparison of predictive performances of logistic regression and causal Bayesian network.

Indice	Logistic regression	Bayesian network	p-value*
Youden Index	0.36	0.33	/
Sensibility (95% CI)	0.71 (0.65–0.76)	0.70 (0.65–0.75)	/
Specificity (95% CI)	0.65 (0.64–0.66)	0.62 (0.61–0.63)	/
Positive predictive value (95% CI)	0.08 (0.07–0.09)	0.07 (0.06–0.08)	/
Negative predictive value (95% CI)	0.98 (0.978–0.986)	0.98 (0.977–0.985)	/
Positive Likelihood Ratio (95% CI)	2.05 (1.89–2.22)	1.87 (1.72–2.02)	/
Negative Likelihood Ratio (95% CI)	0.44 (0.37–0.43)	0.47 (0.39–0.57)	/
Area under curve (95% CI)	0.72 (0.70–0.75)	0.71 (0.68–0.73)	0.06

* Test of contrast between ROC curves,

p>0.05 means no statistical differences between area under curves.

**Fig 2:**
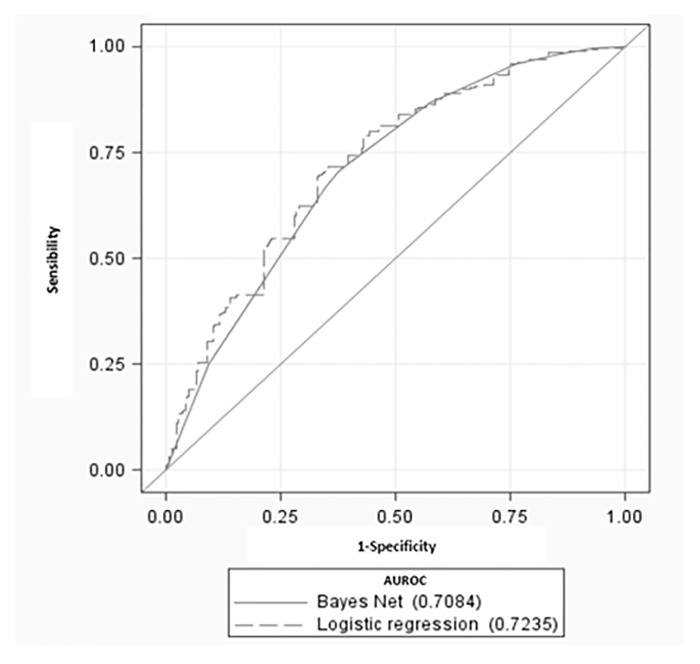
Comparison of ROC curves for each model.

The logistic regression analysis found a statistically significant association of fracture with several variables, including sedative or anxiolytics use, Age, BMD, BMI, gait speed, personal history of fracture and history of more than 2 falls in the previous semester. All relationships described below were adjusted upon these variables. Regarding BMI, having a BMI higher than 30 appears to have a protective effect (adjusted Odds Ratio (aOR) = 0.64 (95% CI: 0.42–0.98)), contrasting with the trend observed for women having a BMI below 18.5 (aOR = 1.67 (0.98–2.87)). Regarding gait speed, we observed that the higher the measured gait speed, the less the patient was prone to sustain a fracture, suggesting an important effect of gait over the fracture risk. The 5TSTS test results were not retained during the backward approach when both gait speed and 5TSTS where included. Presence of a personal history of fracture (aOR = 1.39 (1.06–1.80)) and of a number of fall greater than two in the past six months (aOR = 1.86 (1.06–3.04)) were positively associated with sustaining a hip fracture in the next four years. Current use of sedative or anxiolytics was associated with an increase of hip fractures risk (aOR = 1.32 (1.02–1.69)). The Area Under Receiver Operator Curve (AUROC) was 0.72 (0.70–0.75), not statistically different from that obtained with the Bayesian network. These are plotted in Fig 2.

A significant interaction between psychotropic drugs and age, between psychotropic drugs and Bone Mineral Density, between Age and gait speed and between age and number of fall in the past six months was detected during the logistic regression modeling process. In order to account for interaction, four logistic regression models were developed in each strata of psychotrope use (user and non-users) and age (<80, 80-<85, 85-<90, > = 90 years, data not shown). The stratification on psychotropic drug use showed that in the group of psychotropic non-users, aging had a positive association with hip fracture, in contrast with the group of users, where age did not showed a similar association. On the other hand, association of low bone mineral density were far stronger in the psychotropic drug users group (aOR = 7.93 (3.70–17.2)) than in the non-user group (aOR = 1.63 (0.94–2.84)).

## Discussion

Our results showed that age, gait speed and BMD were the variables having the greatest influence on hip fracture occurrence in both modeling approach. Furthermore, according to the *a priori* causal assumptions encoded in the Bayesian network representation, gait speed and bone mineral densities are suspected to mediate effect of all other observed variables, including age.

A recent study involving causal effect estimation of bazedoxifene acetate on fracture by use of structural equation modeling found age and body mass index to be causally linked to BMD that, in turn, had an effect on fractures [25]. Our results suggest that gait-speed is directly associated with fracture and mediates the effect of other variables. We did not retrieve another study focusing directly the relationship between gait-speed and fracture. Most of the papers focused on the link between falls and gait-speed, falls being now identified as a direct consequence of gait-speed impairment [26]. In our analysis, the gait speed variable may have subsumed both gait speed impairment and the related falls leading to fractures occurrence. A study focused on this specific research question is necessary. However, these observations suggest that an optimal prevention of hip fracture has to be thought as a multi-component intervention, at least involving preservation of structural properties of bone and improvement of gait in the meantime.

Graphical algorithmic approach presented at least two contributions to more traditional regression analysis. First, the use a graphical representation highly facilitated interaction and comprehension between the medical expert and the modeler [27]. Second, the graph can be used to deal more efficiently with confounding factors than in traditional multivariate regression using stepwise algorithm [28] (despite that we didn’t used this ability in the present work).

In our work, logistic regression and the causal Bayesian network have highlighted same variables as most influential upon the fracture process. Moreover, both approaches showed themselves complementary, as logistic regression permitted us to detect easily interactions of psychotropic drug use with age and BMD, which was more difficult by use of Bayesian network alone. We observed that the effect of low BMD was far stronger in the population using psychotropic drugs. The mechanism suggested by the Bayesian network analysis involves a degradation of gait, in turn directly linked to fall history recorded in the six month before the study started. Other data supports this hypothesis. A recent study involving the synthesis of the results of 160 studies available from the literature in a single Bayesian network and the evaluation of its predictive performances on a sample of 288 institutionalized elderly patients found that psychotropic drugs was also a predictive factor for fall [29], which is in turn strongly and causally associated to hip-fracture.

We didn’t account for time and for competitive risk. However, the initial analysis of EPIDOS data using Cox models and accounting for time found also a great influence of gait-speed and BMD in the occurrence of hip fracture [15] and some other study involving more traditional analysis accounting for time showed similar results [30–33]. Incorporation of time, competitive risk and hierarchical structures embedded in the data in Bayesian network modeling is possible an represent an important field of research in bioinformatics [34,35]. This point is a future development of the current work.

Another point which could be discussed is the prior assumptions used in our Bayesian network learning phase. These were based on the logical definition of the manipulability theory [36,37]. This theory states that x may influence y only in the case that one can change x in order to change y. For instance, ageing can change the health status of the patient, for example by causing osteoporosis, but this relationship is not reciprocal as having osteoporosis does not change age of the patient. One of the limits of our approaches is that causal Bayesian network is very sensitive to causal assumption misspecifications. Despite the fact that our prior assumptions were the most objective as possible, they may nonetheless represent solely the experience or opinion of the experts involved in the project. However, these assumptions are made transparent and easily understandable for the reader willing to criticize them, which is not always the case in other framework [38]. Thus, it is clear that our results must be considered as hypotheses, which could evolve with knowledge’s updates in the field according to experimental data.

Finally, there may be residual confounding, which may appear when major variable causing the studied event are omitted from the analysis. However, such residual confounding is a flaw that could threaten all statistical analysis of epidemiological data and is not specific to causal Bayesian network framework.

## Conclusion

Causal Bayesian network and logistic regression were both shown that age, gait speed and BMD were the variables having the most noticeable influence on hip fracture occurrence. Moreover, BN suggested that they could mediate effects of all other major risk factors. These observations give an insight on the complexity of the hip fracture event and suggest that both objectives, i.e. improvement in mobility of the patient and bone structure preservation, must be encompassed in the same therapeutic management to efficiently prevent hip fractures.

## Supporting Information

S1 Dataset Hip fractures during the EPIDOS Study. This database contains all the information on which the analyzes were performed.
(XLS)
